# Prevalence of MRSA nasal carriage among pregnant women in Copenhagen

**DOI:** 10.1371/journal.pone.0246343

**Published:** 2021-01-29

**Authors:** Mona Katrine Alberthe Holm, Thilde Nordmann Winther, Sisse Kammann, Marianne Skovby Rasmusson, Lis Brooks, Henrik Westh, Mette Damkjær Bartels

**Affiliations:** 1 Department of Clinical Microbiology, Copenhagen University Hospital Hvidovre, Hvidovre, Denmark; 2 Department of Pediatrics and Adolescent Medicine, Copenhagen University Hospital Hvidovre, Hvidovre, Denmark; 3 Department of Obstetrics and Gynecology, Copenhagen University Hospital Hvidovre, Hvidovre, Denmark; 4 Faculty of Health and Medical Sciences, Institute of Clinical Medicine, University of Copenhagen, Copenhagen, Denmark; Instituto de Technologia Quimica e Biologica, PORTUGAL

## Abstract

**Background:**

Methicillin resistant *Staphylococcus aureus* (MRSA) frequently causes outbreaks in neonatal intensive care units (NICUs). It is believed that MRSA predominantly enters the NICU with MRSA colonized parents. In Denmark, 27 MRSA NICU outbreaks have been registered between 2008 and 2019.

**Aim:**

The aim of this study was to determine the prevalence of MRSA nasal carriage in pregnant women in Copenhagen and to clarify if MRSA screening during pregnancy could add to the prevention of NICU outbreaks.

**Methods:**

All pregnant women 18 years or older were offered MRSA nasal screening at their first midwife visit between 13 and 20 weeks of gestation.

**Results:**

1778 pregnant women were included, two (0.11%) carried MRSA in the nose.

**Conclusion:**

Infants of the two MRSA positive women were not admitted to a NICU and therefore the screening had no impact on NICU outbreaks. The low prevalence of MRSA found in this study does not justify MRSA screening of all pregnant women in Denmark.

## Introduction

Methicillin resistant *Staphylococcus aureus* (MRSA) is a common pathogen in maternal post-partum infections [1] and frequently causes outbreaks in neonatal intensive care units (NICUs) [2–4]. Transmission of MRSA to neonates probably occurs most often from asymptomatic parents, however, transmission from healthcare workers, siblings, visitors and environmental surfaces is also well documented [5–8]. Caesarian section, use of nasal continuous positive airway pressure (nCPAP) [9], low birth weight, skin to skin contact and length of stay in the NICU are risk factors significantly associated with MRSA colonization or infection [8–10].

Denmark has a low MRSA prevalence with 3657 new cases in 2019, a number which has quadrupled within the last decade [11]. In 2019, 49.0% of all MRSA cases were community acquired (CA-MRSA) [11] and asymptomatic carriage is often found. The MRSA prevalence within different age groups in our catchment area is highest among children between 0–4 years, and adults between 25–39 years of age (unpublished data), the latter being the typical age of pregnant women and their partners. The increasing number of mainly CA-MRSA in this age group is worrying, as it is hypothesized that the MRSA outbreak strain usually enters the NICU with a colonized parent [3]. A total of 27 MRSA outbreaks have been registered at the 17 Danish NICUs in the period 2008 to 2019. The outbreaks comprised of a total of 554 MRSA positive persons, each outbreak consisting of three to 85 neonates, parents and health care workers, with some outbreaks spreading over several years.

Hvidovre Hospital is situated in the Capital Region of Denmark and has the largest maternity ward in the country with more than 7000 births per year [12] accounting for 11.4% of all births in Denmark in 2018 [13]. Approximately 10% of all neonates born in Denmark receive shorter or longer term NICU care [14].

It has been suggested that MRSA admission screening, especially in high risk settings such as intensive care units, could be useful for early recognition of asymptomatic MRSA carriage and outbreak prevention [3, 15, 16]. The aim of this study was to determine the prevalence of MRSA nasal carriage in pregnant women within the Hvidovre Hospital catchment area and to clarify if MRSA screening during pregnancy could prevent NICU outbreaks.

## Material and methods

From May 18^th^ to September 25^th^, 2015 all pregnant women, 18 years and older, were offered MRSA nasal screening at their first midwife visit, between 13 and 20 weeks of gestation at the midwife clinics of Hvidovre Hospital. To obtain reliable insight in the MRSA prevalence we determined that a > 3-month screening period would be representative. The aim was to include 1500 pregnant women in the study.

### Recruitment

Along with the first midwife invitation all women received an additional letter about the possibility of entering the study. The MRSA nasal screening was performed by the pregnant woman herself at the first midwife visit. Posters in the bathrooms of consultation clinics showed how to perform the swab correctly from both nostrils. All samples were collected daily by the midwife and send to the Department of Clinical Microbiology, Hvidovre Hospital for MRSA analysis. The women were informed that they would only be contacted by phone in case of a positive MRSA result. In this case, the MRSA Knowledge Center, situated at the Department of Clinical Microbiology, Hvidovre Hospital, would create a personalized decolonization plan for the pregnant woman and household members. The MRSA Knowledge Center follows all MRSA positive persons in the catchment area and is responsible for decolonization of MRSA carriers in collaboration with the patient’s general practitioner.

### Swab handling

Eswabs (Copan) were inoculated in a standardized enrichment broth [17] containing 2.5% NaCl, 3.5 mg/L cefoxitin, and 20 mg/L aztreonam for overnight incubation at 35 °C. From the broth, 10 μL was spread on a 5% blood agar and a MRSA chrome agar (Biomérieux). Susceptibility testing was performed by disk diffusion using EUCAST breakpoints. Isolates were confirmed to be MRSA by an in-house PCR [18]. If MRSA was confirmed, the isolate was routinely whole genome sequenced on an Illumina MiSeq [18] and added to our local database with more than 7000 whole genome sequenced MRSA genomes. In order to determine whether the isolates were connected to other known MRSA isolates in our database, cgMLST analysis was performed using SeqSphere software [19].

#### Ethics

The study was approved by the Hvidovre Hospital Board. There were no risks for study subjects accepting participation and participation was voluntary.

## Results

A total of 2464 pregnant woman attended the midwife clinics during the 18-week study period. Of these, 1778 (72%) accepted participation. MRSA was cultured from 2 women (0.11%). The two MRSA nasal carriers had no known risk factors for MRSA and would not have been screened routinely for MRSA prior to delivery. Prior to decolonization treatment both women were screened from the nose, throat and perineum according to the Danish national guidelines. One was only positive in the nose and was MRSA free after one treatment, while the other woman was nose and throat positive and remained throat positive until after delivery. None of them were admitted to NICU with their infant.

Whole Genome Sequencing (WGS) showed one t127/ST1, Panton-Valentine Leucocidin (PVL) positive isolate and one t105/ST5, PVL positive isolate. Search through our local WGS database, with more than 7000 MRSA genomes from the Copenhagen area, revealed a total of six t127/ST1, PVL positive isolates and four t105/ST5, PVL positive isolates found in 2015. However, cgMLST showed only a single related isolate from a household member for each of the two cases.

## Discussion

Out of 1778 pregnant women self-screened for nasal MRSA carriage at first midwife visit, only two (0.11%) were MRSA positive. As screening was only performed during a four-month period and around 7000 women give birth each year in our catchment area, based on these data we would expect the prevalence to be approximately eight per year. Aside from the two cases recognized during this study, the MRSA Knowledge Center was aware of additionally four pregnant MRSA carriers in 2015 that were not found during this study, but were either known MRSA carriers (1/4), screened by their general practitioner due to MRSA risk factors (2/4) or MRSA found accidentally in a skin sample (1/4). A recent study [20], including 5117 subjects from Danish emergency departments, with the aim of determining the carriage prevalence of resistant pathogens upon admittance, report a 0.3% MRSA prevalence. Their prevalence is slightly higher than what we found, however, in their study patients were screened from rectum, nose and throat, which would most likely catch more MRSA carriers, as we only screened from the nose.

In Denmark, MRSA screening is usually only performed upon hospital admission if risk factors for MRSA carriage are present. Risk factors include regular pig or mink contact, treatment received in hospitals or clinics outside the Nordic countries within the last 6 months, and if patient or household members currently are or previously have been MRSA positive [21]. A trial, performed in Holland, another low MRSA prevalence country, with the aim of determining the prevalence of MRSA nasal carriage, between 2010–2017 [22], performed pre-operative, nasal MRSA swab screening on 30,718 included cases of which 41 cases were MRSA nasal positive (0.13%). Out of the MRSA nasal positive cases 73.2% had no known risk factors for MRSA carriage. The lack of present risk factors in most of the MRSA positive cases from Holland, question the effectiveness of asking only for MRSA specific risk factors upon hospital admission.

When transferring infants between NICUs in Denmark MRSA screening is performed upon arrival. Nevertheless, in some cases the same MRSA clone manages to spread throughout multiple NICUs, and although the yearly number of NICU MRSA outbreaks has remained relatively stable since 2008, there is a tendency towards an increasing number of outbreaks. To reduce spread, targeted MRSA screening in high-risk departments such as NICUs as proposed by Kristinsdottir et. al could be considered [3]. As approximately 10% of all neonates in Denmark are admitted to NICU, routine MRSA screening of either mother or infant upon NICU admission and not just when transferred between NICUs would amount to an additionally 6000 admissions screenings each year. While screening upon NICU admission reduces the number of NICU MRSA outbreaks, outbreaks will not be eliminated completely, e.g. due to other family members, visitors and hospital staff introducing MRSA to the ward. Both Kristinsdottir et. al. [3] and Bozzella et al. [23] emphasize the possibility of earlier recognition of possible outbreaks if systematic screenings being introduced, e.g. weekly testing, especially in long term NICU stays. This suggestion could be valuable, but more cost-benefit research is needed on the subject. The advantages of early MRSA screening during pregnancy include the possibility of decolonization prior to delivery, optimization of antibiotic prophylactics during caesarian section and isolation of mother and infant during the entire hospital stay, if MRSA positive. When screening for MRSA between week 13 to 20 of gestation, there is a risk of catching MRSA between the screening and delivery, however, in a low MRSA prevalence country we consider this risk to be very low.

There are limitations to this study. Although we consider a 72% participation rate satisfactory, 28% of the pregnant women did not participate. We speculate that one reason could be that the invitation and sampling instruction was written in Danish which eliminated the possibility of non-Danish speaking women to be included. However, we have no data on who the women were or why they didn’t participate. It is unknown if they had a higher risk of MRSA carriage than the participating women. If they had recently lived in refugee camps, which are considered a risk for MRSA carriage [24, 25], or in a country with a higher MRSA prevalence than in Denmark, the true prevalence might be higher than estimated. Another limitation is that the nose swabbing was performed by the pregnant women themselves and possibly have been performed poorly in some cases leading to a lower number of true MRSA positives.

## Conclusion

Due to the very low prevalence of 0.11% MRSA nasal carriage found among pregnant women in this study, we do not recommend screening of all pregnant women in our setting. As NICU MRSA outbreaks are often long-lasting, extremely resource demanding and concern causing for families involved, we believe that focused screening in NICUs could be considered but the best strategy to reduce the number of outbreaks in a low prevalence area remains to be found.

## References

[1] Parriott AM, Chow ALP, Arah OA. Inadequate research on methicillin-resistant Staphylococcus aureus risk among postpartum women. Expert Review of Anti-Infective Therapy. 2013 10.1586/14787210.2013.850027 24151831 PMC5612403

[2] Carey AJ, Duchon J, Della-Latta P, Saiman L. The epidemiology of methicillin-susceptible and methicillin-resistant Staphylococcus aureus in a neonatal intensive care unit, 2000–2007. J Perinatol. 2010 10.1038/jp.2009.119 19710681

[3] Kristinsdottir I, Haraldsson A, Thorkelsson T, Haraldsson G, Kristinsson KG, Larsen J, et al MRSA outbreak in a tertiary neonatal intensive care unit in Iceland. Infect Dis (Auckl). 2019 10.1080/23744235.2019.1662083 31507231

[4] Pierce R, Lessler J, Popoola VO, Milstone AM. MRSA acquisition risk in an endemic NICU setting with an active surveillance culture and decolonization program. J Hosp Infect. 2017 10.1016/j.jhin.2016.10.022 PMC5219853 27887754

[5] Andersen BM, Lindemann R, Bergh K, Nesheim BI, Syversen G, Solheim N, et al Spread of methicillin-resistant Staphylococcus aureus in a neonatal intensive unit associated with understaffing, overcrowding and mixing of patients. J Hosp Infect. 2002 10.1053/jhin.2001.1128 11825047

[6] Geva A, Wright SB, Baldini LM, Smallcomb JA, Safran C, Gray JE. Spread of methicillin-resistant Staphylococcus aureus in a large tertiary NICU: Network analysis. Pediatrics. 2011 10.1542/peds.2010-2562 PMC3208963 22007011

[7] Eveillard M, Martin Y, Hidri N, Boussougant Y, Joly-Guillou M-L. Carriage of Methicillin-Resistant Staphylococcus aureus Among Hospital Employees: Prevalence, Duration, and Transmission to Households. Infect Control Hosp Epidemiol. 2004 10.1086/502360 14994935

[8] Cheng VCC, Wong SC, Cao H, Chen JHK, So SYC, Wong SCY, et al Whole-genome sequencing data-based modeling for the investigation of an outbreak of community-associated methicillin-resistant Staphylococcus aureus in a neonatal intensive care unit in Hong Kong. Eur J Clin Microbiol Infect Dis. 2019 10.1007/s10096-018-03458-y 30680562

[9] Ramsing BGU, Arpi M, Andersen EA, Knabe N, Mogensen D, Buhl D, et al First Outbreak with MRSA in a Danish Neonatal Intensive Care Unit: Risk Factors and Control Procedures. PLoS One. 2013 10.1371/journal.pone.0066904 PMC3692537 23825581

[10] Sakaki H, Nishioka M, Kanda K, Takahashi Y. An investigation of the risk factors for infection with methicillin-resistant Staphylococcus aureus among patients in a neonatal intensive care unit. Am J Infect Control. 2009 10.1016/j.ajic.2009.02.008 19535174

[11] SSI. No Title [Internet]. MRSA—opgørelse over sygdomsforekomst 2019. 2020 [cited 2020 Sep 1]. https://www.ssi.dk/sygdomme-beredskab-og-forskning/sygdomsovervaagning/m/mrsa-2019

[12] foedeafdelingen @ www.hvidovrehospital.dk [Internet]. https://www.hvidovrehospital.dk/afdelinger-og-klinikker/foedeomraadet/Sider/foedeafdelingen.aspx

[13] Statistik D. Denmark birth statistiscs [Internet]. 2018 [cited 2020 Sep 1]. https://www.dst.dk/da/Statistik/emner/befolkning-og-valg/foedsler/foedsler

[14] Klinisk, Epidemiologisk epidemiolog og datamanager fra R har stået for udarbejdelse af analyser og, Kommentering. Dansk Kvalitetsdatabase for Nyfødte (DKN) National årsrapport 2018 [Internet]. 2018. https://www.sundhed.dk/content/cms/73/102373_2019-11-05-dkn_aarsrapport_2018_offentlig-version.pdf

[15] Giuffrè M, Cipolla D, Bonura C, Geraci DM, Aleo A, Di Noto S, et al Epidemic spread of ST1-MRSA-IVa in a neonatal intensive care unit, Italy. BMC Pediatr. 2012 10.1186/1471-2431-12-64 PMC3407518 22682025

[16] Rocha Luis E. C., corresponding author, Singh Vikramjit, Esch Markus, Lenaerts Tom, Liljeros Fredrik and T A. Dynamic contact networks of patients and MRSA spread in hospitals. Sci Rep. 2020;Published online 2020 Jun 9. 10.1038/s41598-020-66270-9 32518310 PMC7283340

[17] Böcher S, Middendorf B, Westh H, Mellmann A, Becker K, Skov R, et al Semi-selective broth improves screening for methicillin-resistant Staphylococcus aureus. J Antimicrob Chemother. 2010 10.1093/jac/dkq001 20130023

[18] Bartels MD, Larner-Svensson H, Meiniche H, Kristoffersen K, Schønning K, Nielsen JB, et al Monitoring meticillin resistant Staphylococcus aureus and its spread in Copenhagen, Denmark, 2013, through routine whole genome sequencing. Eurosurveillance. 2015;20(17). 10.2807/1560-7917.es2015.20.17.21112 25955776

[19] Jünemann S, Sedlazeck FJ, Prior K, Albersmeier A, John U, Kalinowski J, et al Updating benchtop sequencing performance comparison. Nature Biotechnology. 2013 10.1038/nbt.2522 23563421

[20] Skjøt-Arkil H, Mogensen CB, Lassen AT, Johansen IS, Chen M, Petersen P, et al Carrier prevalence and risk factors for colonisation of multiresistant bacteria in Danish emergency departments: A cross-sectional survey. BMJ Open. 2019 10.1136/bmjopen-2019-029000 31253624 PMC6609076

[21] Authority DH. Guidance on Preventing the Spread of MRSA [Internet]. December 13. 2016. p. ISBN online: 978-87-7104-854-4. https://www.sst.dk/da/sygdom-og-behandling/smitsomme-sygdomme/mrsa/~/media/F3F52EC1C6A94C6080F50F435DA02E59.ashx

[22] Weterings V, Veenemans J, van Rijen M, Kluytmans J. Prevalence of nasal carriage of methicillin-resistant Staphylococcus aureus in patients at hospital admission in The Netherlands, 2010–2017: an observational study. Clin Microbiol Infect. 2019 10.1016/j.cmi.2019.03.012 30928560

[23] Bozzella MJ, Soghier L, Harris T, Zell L, Short B Lou, Song X. Impact of decolonization on methicillin-resistant Staphylococcus aureus transmission and infection in a neonatal intensive care unit. Infect Control Hosp Epidemiol. 2019 10.1017/ice.2019.217 31362800

[24] Rasheed NA, Hussein NR. Methicillin-resistant Staphylococcus aureus carriage rate and molecular characterization of the staphylococcal cassette chromosome mec among Syrian refugees in Iraq. Int J Infect Dis. 2020 10.1016/j.ijid.2019.12.006 31843670

[25] Reinheimer C, Abdollahi P, Zacharowski K, Meybohm P, Mutlak H, Klingebiel T, et al Prevalence of multidrug-resistant organisms in refugee patients admitted to a German university hospital depending on duration of stay in Germany. GMS Hyg Infect Control. 2019 10.3205/dgkh000323 31293878 PMC6606948

